# Affiliation with Natural Products at KIB of Prof. Zhou Jun: On the Occasion of 80th Anniversary of Kunming Institute of Botany, CAS

**DOI:** 10.1007/s13659-018-0183-9

**Published:** 2018-07-16

**Authors:** Jiang-Miao Hu

**Affiliations:** 1 State Key Laboratory of Phytochemistry and Plant Resources in West China, Kunming Institute of Botany, Chinese Academy of Sciences, Kunming, 650201 People’s Republic of China; 2 Yunnan Key Laboratory of Natural Medicinal Chemistry, Kunming Institute of Botany, Chinese Academy of Sciences, Kunming, 650201 People’s Republic of China

**Keywords:** Prof. Zhou Jun, Plant resources, *Panax*, *Paris*, *Gastrodia*, *Cynanchum*

## Abstract

Prof. Zhou Jun, Academician of Chinese Academy of Sciences (1999), is a phytochemist and medicinal chemist of China. He is one of the pioneers of Kunming Institute of Botany, CAS and a major founder of the State Key Laboratory of Phytochemistry and Plant Resources in West China. The chemical compositions of some TCM from genus of *Dioscorea*, *Aconitum*, *Panax*, *Paris*, *Cynanchum*, *Gastrodia*, *Dendrobium* etc. and family Asclepiadaceae, Caryophyllaceae, Hypoxidaceae etc. have been explored by Prof. Zhou′s team as steroids, triterpenoids, alkaloids, cyclic peptides and phenols etc., which revealed the main active composition of those TCM such as *Panax notoginseng*, *Paris yunnanensis* and *Gastrodia elata*.

## Growth and Work Experience

**Fig. 1:**
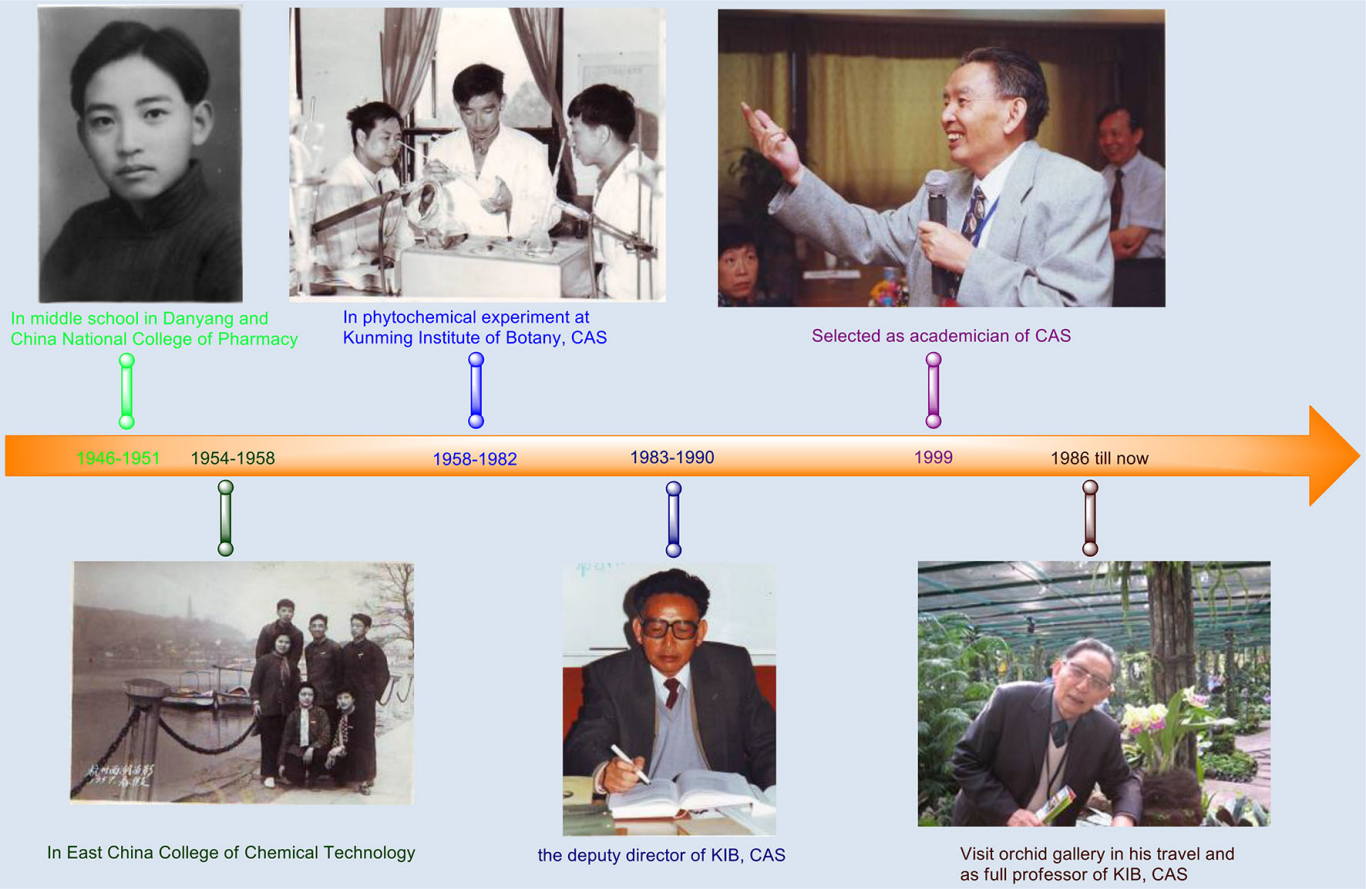
Timeline of Prof. Zhou Jun from 1946 to present

Prof. Zhou Jun was born on February 5, 1932 in Dongtai County, Jiangsu Province in a family of teachers (two generations, grandfather and uncle, successively acting as teachers in rural private school). Under the influence of the family tradition and his own learning hobby, the destitute family strived laboriously to provide education opportunities for Prof. Zhou in his childhood. From the countryside private school to middle school in Danyang (1946), then to China National College of Pharmacy (Nanjing, Predecessor of China Pharmaceutical University) in 1948, Prof. Zhou started his affiliation with natural products since his undergraduate years. The manuscript described here is dedicated to celebrating the opportunity of 80th anniversary of Kunming Institute of Botany, Chinese Academy of Sciences (CAS), and 70th years of Prof. Zhou′s career (Fig. 1).

After graduating from the National College of Pharmacy, Prof. Zhou worked as a Drug Manager in Nanjing Medical Corps which supported North Korean. Later, he came back from the northeast of China and took part in the prepare for construction of East China College of Pharmacy (Nanjing, Predecessor of China Pharmaceutical University) and then served in Health Bureau of east China in Shanghai. To further improve the professionalism, Prof. Zhou started his higher education from 1954 to 1958 in East China College of Chemical Technology (East China University of Science and Technology in now time).

Upon graduating from East China College of Chemical Technology in 1958, Professor Zhou Jun resolutely gave up the job opportunities in Beijing and Shanghai, while responding to the call of the country to support the frontier construction and joined Kunming workstation of Institute of Botany, CAS. Since he came to Kunming, he was assigned to the newly-established group which focused on phytochemical resource with only eight members. At the end of the year, Zhou Jun served as the academic secretary of the group. The workstation was then scaled up to a research institute at the end of 1959, and the phytochemical resource group was postponed and renamed as the Wild Plant Resource Research Laboratory and later as the Phytochemistry Research Laboratory in 1963.

Since the entry into Kunming station, Zhou Jun has been promoted to the actual academic leader of this Laboratory from the founder of the lab under the leadership of botanists, Prof. Cai Xi-Tao and Prof. Wu Zheng-Yi. Prof. Zhou served as a director of Phytochemistry Laboratory from 1976 to 1982, deputy director (1980–1982) and director (1983–1990) of Kunming Institute of Botany, CAS. Meanwhile, Zhou Jun was thereafter promoted as an assistant professor in 1962, associate professor in 1978 and full professor in 1986. He was elected as an academician of Chinese Academy of Sciences in 1999.

## Academic Achievements and Social Contributions

**Fig. 2:**
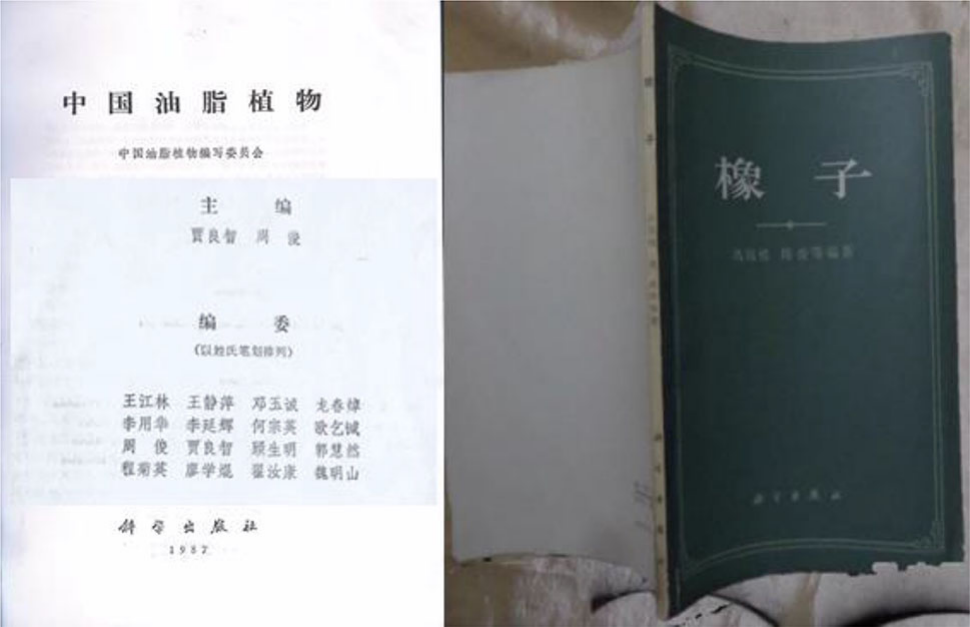
Monographs: “Chinese oil plant (中国油脂植物)” and “Acorns (橡子)”

Over the past 60 years from 1958 to present, Prof. Zhou Jun devoted himself to the field of plant resources in phytochemistry and medicinal chemistry. His interest has been concentrated on chemical and pharmaceutical investigation of plants of *Dioscorea*, *Aconitum*, *Panax*, *Paris*, *Cynanchum*, *Gastrodia*, *Dendrobium*, and some other genus of Fagaceae, Asclepiadaceae, Caryophyllaceae, Hypoxidaceae etc. He and his colleagues have isolated and eluciadated more than 950 compounds, mainly including steroids, triterpenoids, alkaloids, cyclic peptides and phenols, and published over 400 academic papers and edited 2 monographs “Chinese oil plant (中国油脂植物)” and “Acorns (橡子)” (Fig. 2) [1, 2]. By phytochemical research of these herbal medicine and got different types of natural products, Prof. Zhou Jun devoted his lifelong energy to develop medicine from natural source and the initial phytochemical resource group in Kunming workstation of Institute of Botany (CAS) adjusts to the field of medicinal phytochemistry.

### Plant Resources for Pharmaceuticals and Foods

In the early years of his scientific career, he had a special moment with the harsh environment of the frontier, from which Zhou Jun and his colleagues did not shrink. Under the care and support of the State, the CAS and the leadership of the local government of Yunnan Province, Zhou Jun has undertaken national and local scientific research tasks, and has driven the development of disciplines and research institutes with tasks. Accordingly, scientific achievements were adopted to promote the development of national economy, especially to advance the pharmaceutical industrialization of Yunnan Province.

**Fig. 3:**
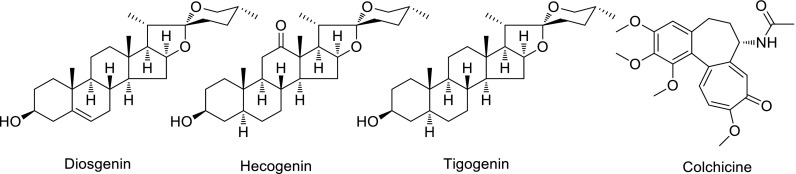
Chemical structure of diosgenin, hecogenin, tigogenin and colchicine

In 1959, for instance, our country needed domestic raw materials urgently for the synthesis of steroid hormones and raw materials for the contraceptive drug. Under the circumstance, Zhou Jun and his colleagues undertook this task. During the period from 1959 to 1963, they completed the research on the chemical composition and distribution of diosgenin, hecogenin and tigogenin (Fig. 3) from domestic plant of Dioscoreaceae and Agavaceae. Then the results were reported that eight species of *Dioscorea* such as *D. zingiberensis* and *D. orbiculata* are ideal raw materials for the synthesis of steroid hormones [3, 4]. From 1962 to 1964, he completed the research on the chemistry and production technology of the domestic colchicine (Fig. 3) from *Iphigenia indica* A. Gray and then put into production by the Kunming Pharmaceutical Factory in 1973 [5].

### Chemical Material Basis of TCM

Traditional Chinese Medicine (TCM) plays a big role in the medical system which is for the purpose of health care and treatment of diseases in China and regarded as treasure for wellbeing. With the concerted efforts of Prof. Zhou and his colleague, large equipment, such as NMR (WH-90), GC-1B, IR-450, UV-210A and GC/MS 4510 were acquired and elucidating platform of natural products was then framed at the time. The direction of Phytochemistry Research Laboratory was then turned to the basic research on chemical composition of TCM and ethno medicine, such as Yunnan Baiyao.

**Fig. 4:**
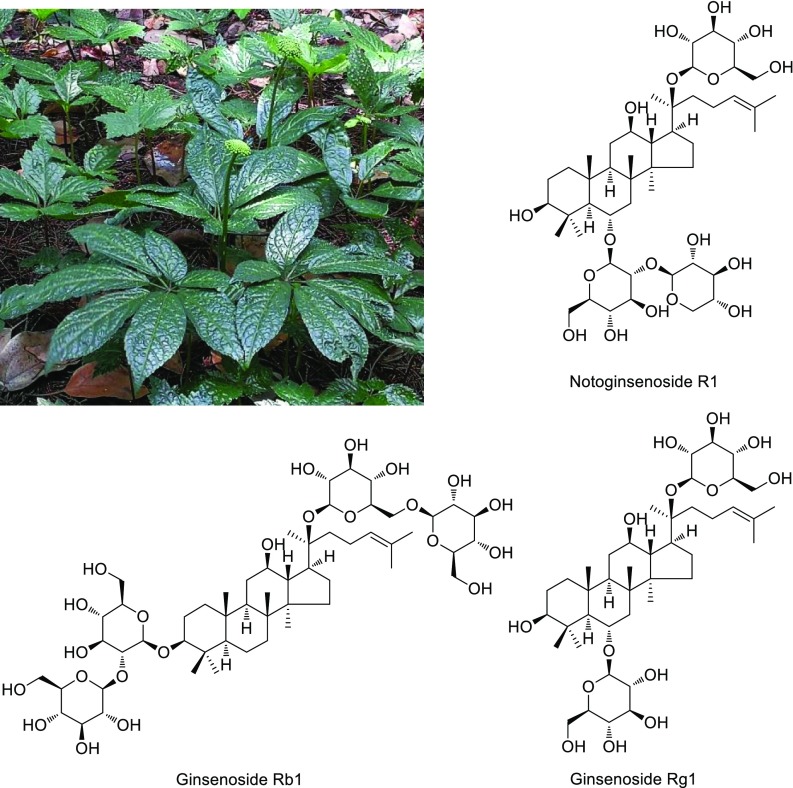
Main ginsenoside from *Panax notoginseng*

In 1970s, for instance, the main medicinal active components of *Panax notoginseng* were identified by Prof. Zhou Jun and his colleague (Fig. 4) [6–12]. Furthermore, after chemical research and comparative analysis focusing on the plants of *Panax* L., the distribution characteristics and variety specificity of triterpenoids and triterpenoid saponins were summarized within the genus *Panax* [13]. The chemical composition of *P. ginseng* and *P. quinquefolium* saponin was chemically revealed for the first time. In plant morphology, the rhizomes of ginseng (*P. ginseng*), American ginseng (*P. quinquefolius*) and *P. notoginseng* are erect rhizomes, and the chemical composition is not affected by the geographical distribution. It clarified the confusion in the research of *Panax* species and promoted the research and development of *Panax* species. This research achievement was adopted by the Pharmacopoeia and the pharmaceutical industry of China.

**Fig. 5:**
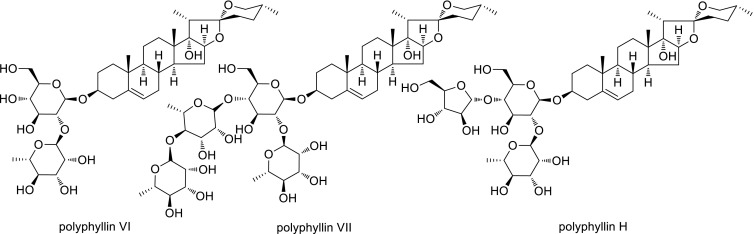
Main bioactive components of polyphyllin VI, VII and H from the rhizome of *Paris*

**Fig. 6:**
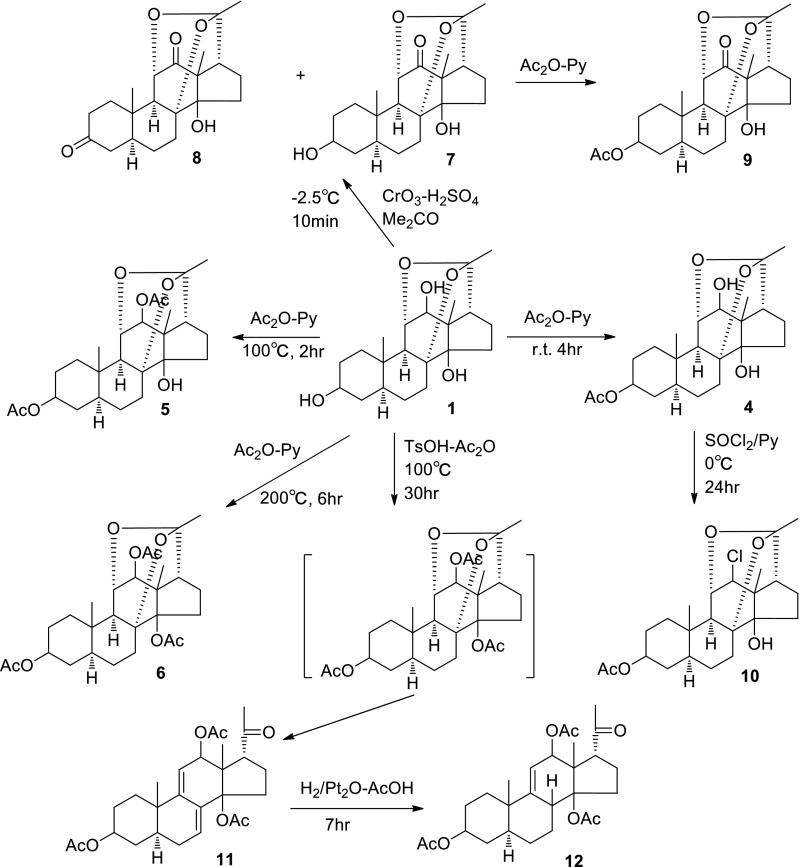
Chemical modification for elucidation of tenacigenin A

After chemical research of *Dioscorea* and acquisition of steroidal saponins, further chemical research of such kind of natural products were undertaken especially with plants of Liliaceae (*Paris*) [14–20] and Asclepiadaceae (*Marsdenia* and *Cynanchum*) [21–31]. Chemical active composition for uterine contraction and stopping bleeding as steroidal polyphyllins (Fig. 5) from *Paris* was then revealed. Stem of the plant *Marsdenia tenacissima* was used as folk medicine for tracheitis and tumor in Yunnan province. After a series of chemical modifications such as hydrolysis, oxidation, acetylation, halogenation and hydrogenation etc. and combined with physical analysis, the main composition of the plant, a C21-steroidal sapogenin was then elucidated and named as tenacigenin A (compound **1** in Fig. 6).

**Fig. 7:**
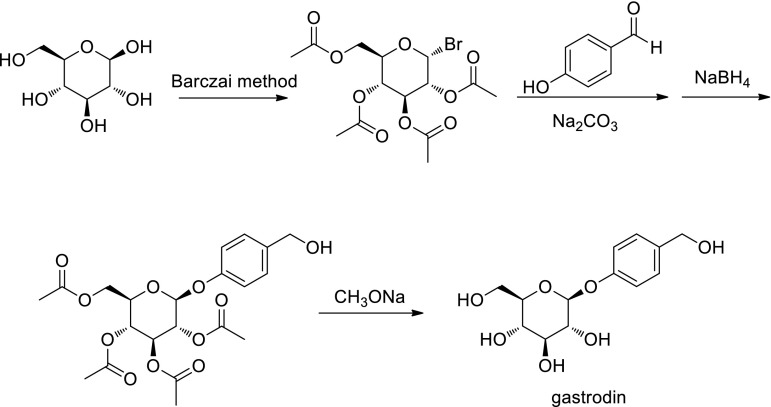
Total synthesis route of gastrodin at the time

From 1978 to 1980, Zhou Jun, Yang Yanbin, and Yang Chongren reported the chemical composition of *Gastrodia elata* Bl. and found the active substance basis of the TCM "Tianma" [32–35]. Furthermore, the synthesis of gastrodin was then performed (Fig. 7) by them and the application of synthetic gastrodin has been applied so far [36].

Chemical synthesis of gastrodin (Tianmasu, originally identified from *Gastrodia elata*) was then industrialized as a therapeutic agent of migraine and neurasthenia, together with the industrialization of *Panax notoginseng* (Xuesaitong), *Paris* spp. (Gong-Xue-Ning, the main components with uterine contraction and hemostasis activity of polyphyllin VI, VII and H were discovered, see Fig. 5), *Dioscorea* spp. and so on from the research assistant of Zhou Jun and his colleagues by the pharmaceutical companies has made huge economic benefits.

### Plant Cyclopeptides and Natural Phenols

The Third Plenary Session of the 11th Central Committee Congress has ushered in a new era of science and technology. Professor Zhou Jun and his colleagues took advantage of golden opportunity of reform and opening up, strengthened external communication and exchanges, and initiated the construction and development of the discipline of the plantation room at the time. Great efforts have been made since then to improve the conditions for the experimental facilities and a large number of equipment has been purchased for natural products isolation and elucidation platform.

**Fig. 8:**
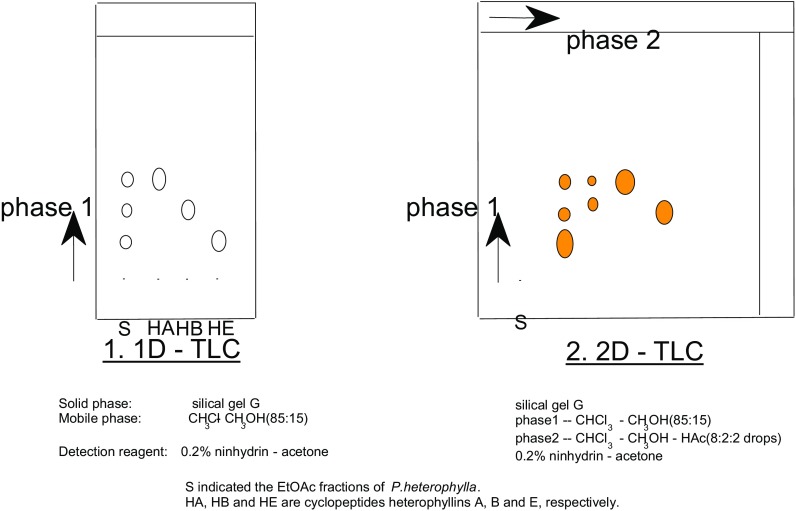
Application of a new TLC method for detection of cyclopeptides in plants

**Fig. 9:**
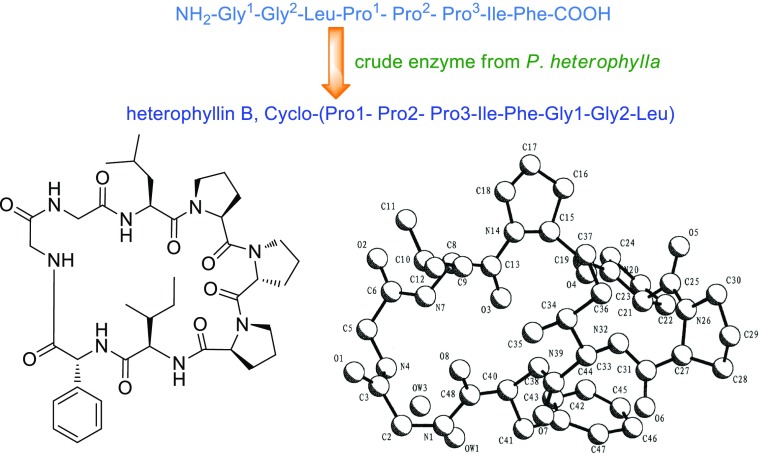
Cyclization of heterophyllin B by cyclase from *P. heterophylla*

Since then (1991), Prof. Zhou and his group have mainly focused on the chemical research of plant cyclopeptides from some plants of Caryophyllaceae and Rubiaceae [37–50]. More than 100 plant cyclic peptides have been elucidated from 28 plant material in Rubiaceae etc. Afterwards, new detection methods of cyclic peptide (Fig. 8) [51], classification [50], synthesis (Fig. 9) [52] and bioactivity have been developed by Prof. Zhou and his colleague [11]. In recent years, natural active phenolic ingredients from some plants of Gymnosperm, Hypoxidaceae and *Dendrobium* become the major interest object of Prof. Zhou Jun [53–60].

### Theoretical Innovation and Discipline Drive

He has developed great insight in summarizing the scientific law from the research experiments to construct systemically innovative theory. He has revolutionarily combined his earlier research in phytochemistry with plant phylogenetic relationship and geographic distribution to explore the *Panax*, *Acconitum* and *Cynanchum* species [13, 61–63]. Based on his long-term research and scientific understanding on “Yunnan Baiyao”, he has proposed an idea of Natural Combinatorial Chemical Libraries that multi-components of TCM are the basis of TCM and act on multiple targets on human body [64, 65].

Through the long-term and systematic research of TCM such as Yunnan Baiyao, the developments of related fields such as medicinal resources and phytochemistry have been promoted in Kunming Institute of Botany, CAS. For instance, steroidal saponin from *Cynanchum* and *Paris*, diterpenoid alkaloid from *Aconitum*, triterpenoid saponins from *Panax* etc. and related pharmaceutical research have been developed to an international level.

## Achievement as a Leader and Tutor of KIB

Professor Zhou Jun paid attention to the exchanges and cooperation with the outside world during his tenure as Director of the Kunming Institute of Botany, CAS. In the process of academic exchanges, the quality of our employees has been improved including degree and technology level. He has trained over 50 postgraduate with master or doctor degree students till now, together with five students studying for a doctorate following him. More than 20 of his students have become academic leaders who are active in the phytochemical field. As a result of his research work and team building, Zhou Jun has laid the foundation of one important field for the long-term development of Kunming Institute of Botany, CAS. Through the efforts of Prof. Zhou and his colleagues, the Lab of Phytochemistry of Kunming Institute of Botany has become the State Key Laboratory of Phytochemistry and Plant Resource in West China.

## Postscript

As one of Prof. Zhou′s PhD student, I can′t fully describe all achievements and contributions of Prof Zhou in my humble vision with this manuscript alone. All my best wish to Kunming Institute of Botany, CAS on the occasion of her 80th anniversary and sincerely hope she becomes better, together with my best wish to the health of Prof. Zhou.
